# TFEB Dependent Autophagy-Lysosomal Pathway: An Emerging Pharmacological Target in Sepsis

**DOI:** 10.3389/fphar.2021.794298

**Published:** 2021-11-26

**Authors:** Xin Liu, Xinchuan Zheng, Yongling Lu, Qian Chen, Jiang Zheng, Hong Zhou

**Affiliations:** ^1^ Medical Research Center, Southwest Hospital, Army Military Medical University, Chongqing, China; ^2^ Chongqing Institute of Green and Intelligent Technology, Chinese Academy of Sciences, Chongqing, China; ^3^ Key Laboratory of Basic Pharmacology of Ministry of Education and Joint International Research Laboratory of Ethnomedicine of Ministry of Education, Zunyi Medical University, Zunyi, China

**Keywords:** sepsis, TFEB, TFEB activators, autophagy-lysosomal pathway, inflammation, immunity

## Abstract

Sepsis is a life-threatening syndrome induced by aberrant host response towards infection. The autophagy-lysosomal pathway (ALP) plays a fundamental role in maintaining cellular homeostasis and conferring organ protection. However, this pathway is often impaired in sepsis, resulting in dysregulated host response and organ dysfunction. Transcription factor EB (TFEB) is a master modulator of the ALP. TFEB promotes both autophagy and lysosomal biogenesis via transcriptional regulation of target genes bearing the coordinated lysosomal expression and regulation (CLEAR) motif. Recently, increasing evidences have linked TFEB and the TFEB dependent ALP with pathogenetic mechanisms and therapeutic implications in sepsis. Therefore, this review describes the existed knowledge about the mechanisms of TFEB activation in regulating the ALP and the evidences of their protection against sepsis, such as immune modulation and organ protection. In addition, TFEB activators with diversified pharmacological targets are summarized, along with recent advances of their potential therapeutic applications in treating sepsis.

## Introduction

Sepsis is the most common and severe syndrome that can affect a population of critically ill patients (Cecconi et al., 2018). Each year, there are an estimated 48.9 million causes of sepsis globally and 11.0 million sepsis-related deaths (Rudd et al., 2020). The latest definition (“Sepsis-3”) defines sepsis as life-threatening organ dysfunction induced by a dysregulated host response to infection (Cecconi et al., 2018). In this regard, the current pathogenic model refers to sepsis arising from infection. This is followed by the release of pathogen-associated molecular patterns (PAMPs) which induce excessive inflammation, aberrant immunity, and abnormalities in the complement and coagulatory systems; ultimately, these processes can lead to organ dysfunction and life-threatening shock (van der Poll et al., 2017; Cecconi et al., 2018). Although the main route of sepsis has been extensively characterized, there are several new paradigms being investigated with regards to the mechanisms that drive the progression of sepsis, including the disruption of cellular homeostasis and the induction of cell injury and organ damage (Cecconi et al., 2018). For example, there is an accumulating body of evidence indicating that the autophagy-lysosomal pathway (ALP), which plays a fundamental role in cellular homeostasis, antimicrobial immunity and organ protection, is commonly impaired in sepsis, thus resulting in a dysregulated host response and organ dysfunction (Venet and Monneret, 2018; Yin et al., 2019). Previous research has also demonstrated that the restoration or enhancement of autophagy and lysosomal function are highly beneficial and can improve the outcomes of patients with sepsis (Sun et al., 2018; Venet and Monneret, 2018; Ballabio and Bonifacino, 2020). Therefore, the regulation of the ALP may provide us with additional options for the therapeutic intervention of sepsis.

Transcriptional factor EB (TFEB) is a member of the microphthalmia (MiTF/TFE) transcriptional factor family (Irazoqui, 2020). TFEB binds directly to a conserved “GTCACGTGAC” motif, termed as the coordinated lysosomal expression and regulation (CLEAR) element in the promoter regions of targeted genes to upregulate their expression (Pan et al., 2019). Previous work has shown that the CLEAR element is highly enriched in a group of autophagy and lysosomal genes, thereby indicating that TFEB may play a key role in the ALP (Sardiello et al., 2009; Irazoqui, 2020). In addition, emerging evidence has associated TFEB and the TFEB-dependent ALP with pathogenic mechanisms in inflammatory diseases, including sepsis, thus creating the potential for the discovery of new therapeutic options (Jin et al., 2017; Li et al., 2017; Irazoqui, 2020; Ouyang et al., 2021). In this mini-review, we aim to summarize recent advances in the regulatory mechanisms associated with the TFEB- dependent ALP and discuss how the targeting of TFEB may provide us with pharmacological options for the intervention of sepsis.

## The ALP and Sepsis

The ALP mainly consists of the autophagy machinery and its associated lysosomal degradation processes (Martini-Stoica et al., 2016). This pathway is evolutionarily conserved in eukaryotic cells and fundamental for the maintenance of cellular homeostasis under stressful conditions (Levine and Kroemer, 2019). Autophagy is characterized by the formation of intracellular double membrane autophagosomes, which deliver cytoplasmic cargo to the lysosomes for degradation (Yu et al., 2018; Levine and Kroemer, 2019). The formation of autophagosome is mediated by a group of core proteins encoded by the autophagy associated genes (ATG). ATG proteins are assembled as kinase complexes at specific stages to control the initiation, formation and elongation of autophagosomes. Then autophagosomes are fused with lysosomes, in which hydrolases and lysosome-associated proteins mediate the degradation of cargo substances (Ballabio and Bonifacino, 2020). Due to its key role in the sequential mechanisms that allow the clearance of cellular debris, the ALP is indispensable for overcoming metabolic insufficiency, organelle damage, and pathogen invasion. Moreover, defects in the ALP pathway are strongly implicated in numerous disease conditions associated with sepsis, including inflammation, organ injury, and aberrant immunity (Deretic, 2021).

It has been extensively demonstrated that the ALP is beneficial for balancing the immune response and protecting organ function during sepsis. A recent review article described autophagy features extensive crosstalk with innate immune cells, thereby exerting influence on phagocytosis in neutrophils, degranulation in mast cells, along with differentiation and migration in NK cells (Germic et al., 2019). Research has also demonstrated that the activation of autophagy in macrophages can confer protection against sepsis by restoring antimicrobial responses against bacterial infection (Lee et al., 2014; Fang F. et al., 2020) or by restricting the production of inflammatory mediators (Xu et al., 2018; Xia et al., 2019). It has been also widely demonstrated that the induction of autophagy alleviates liver damage (Lin et al., 2014), attenuates pulmonary inflammatory impairment (Nikouee et al., 2021), reduces acute kidney injury (Sunahara et al., 2018), protects against neuromuscular dysfunction (Chen G. et al., 2019) and improves cardiac function (Sun et al., 2018) in preclinical models of sepsis. In contrast, the suppression of autophagy during sepsis has been shown to result in accelerated apoptosis in T cells (Oami et al., 2017), the impairment of phagocytic capacity in microglia (Lee et al., 2019), and the aggravation of acute lung or liver injury (Oami et al., 2018; Liu et al., 2021). Similarly, defects in the degradative function of lysosomes is known to amplify sepsis-induced acute lung injury (Mo et al., 2018) and drive septic cardiac dysfunction (Jia et al., 2018). Moreover, disruption of the fusion between lysosomes and autophagosomes can be induced by excessively low doses of LPS, thus leading to persistent low-grade inflammation in macrophages (Baker et al., 2015). It is notable that the ALP is often disrupted during sepsis; this has been shown to worsen outcomes in preclinical models (Chien et al., 2011) and in clinical populations (Huang et al., 2021). Therefore, targeting the ALP may represent a promising strategy for the intervention of sepsis.

## TFEB is a Central Regulatory HUB of the ALP

It is possible that the induction of initial acute cellular responses by ALP may not involve transcriptional machinery. However, the sustained upregulation of autophagy and lysosomal activity requires modulation by several key transcriptional factors (Levine and Kroemer, 2019). TFEB is a master transcriptional factor of the ALP (Martini-Stoica et al., 2016) and was initially identified as a master regulator of lysosomal biogenesis and degradation (Sardiello et al., 2009). Further studies confirmed that TFEB enhances lysosomal proteostasis of mutated or misfolded proteins (Song et al., 2013), induces a Ca^2+^-dependent exocytosis in the lysosomes (Medina et al., 2011), mediates the clearance of damaged lysosomes, and controls lysosomal membrane repair (Palmieri et al., 2011). Research has also shown that TFEB is an essential regulator at multiple stages of autophagy. For example, TFEB upregulates the expression of several autophagic genes (e.g., *Becn1*, *Gabarap*, and *Maplc3b*) bearing the CLEAR element (Palmieri et al., 2011) while the overexpression of TFEB is known to enhance the biogenesis of autophagosomes in multiple cell lines (Settembre et al., 2011). In addition, TFEB coordinates the fusion of autophagosomes with lysosomes, thereby providing balance in the ALP (Settembre et al., 2011). Collectively, these results suggest that TFEB is a central modulator in the ALP.

**FIGURE 1:**
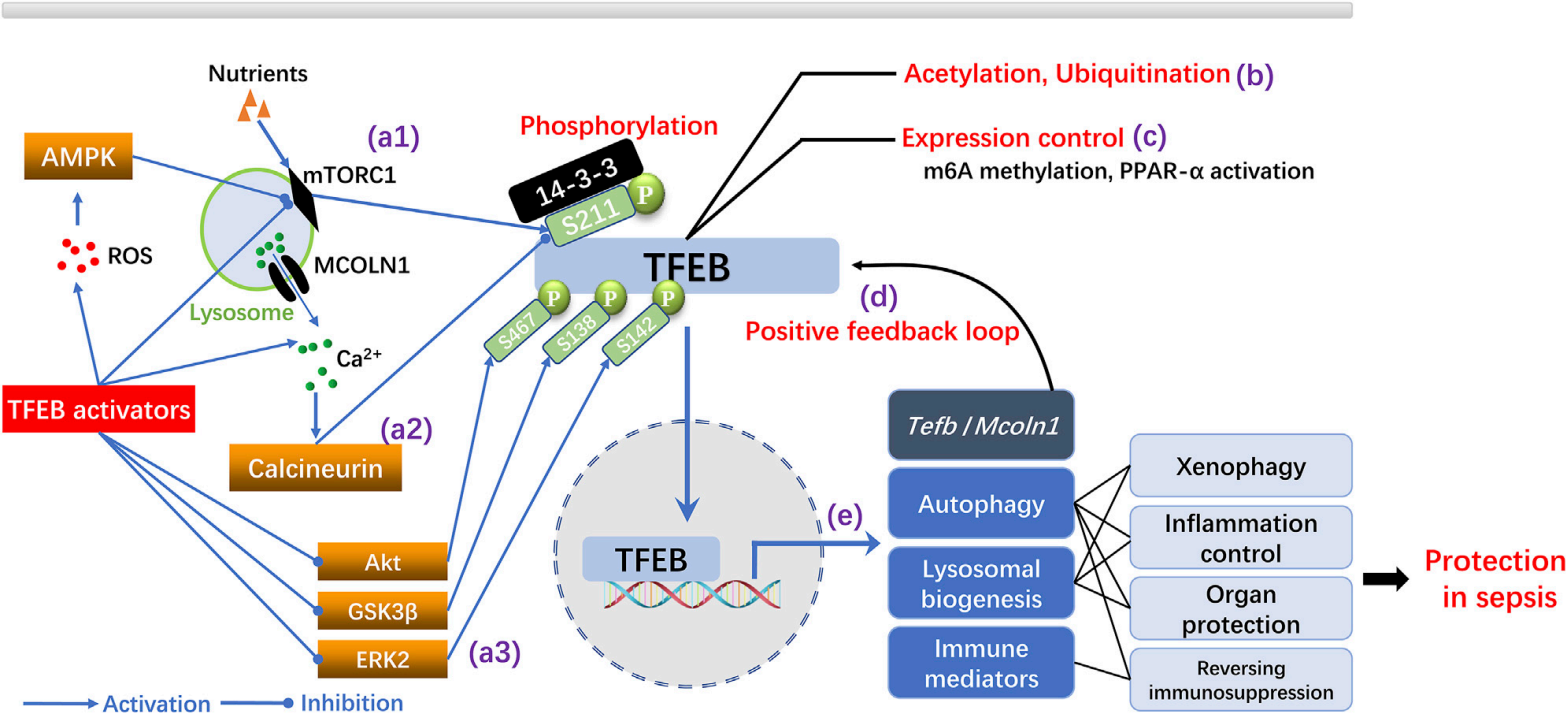
Mechanisms of TFEB activation and the protective roles of TFEB dependent ALP in sepsis. **(A)** Regulation of TFEB activation by phosphorylation. TFEB is inactivated by mTORC1 dependent phosphorylation at S211 while ROS generation and nutrition deprivation inhibit mTORC1 and suppress TFEB phosphorylation (a1). Lysosomal Ca2+ release through the Mcoln-1 channel activate calcineurin and inhibit TFEB phosphorylation (a2). Inhibition of Akt, GSK3β or ERK2 dependent phosphorylation of TFEB promotes TFEB activation (a3) **(B–C)** Modulation of TFEB activation by epigenetic regulation (e.g., acetylation, ubiquitination) or expression control (e.g., m6A methylation, PPAR-α activation). **(D)** Positive feedback regulation of TFEB by transcriptional upregulation of tfeb and mcoln1 by TFEB. **(E)** TFEB upregulates autophagy, promotes lysosomal biogenesis and enhance immunity, thereby conferring protection in sepsis.

The activation of TFEB is mainly regulated by post-translational mechanisms (Zhu et al., 2021) (Figure 1). The mechanistic target of rapamycin complex 1 (mTORC1) and calcineurin are two central modulators that function in an opposing manner to connect upstream pathways with the phosphorylation of TFEB and its cytoplasmic/nuclear distribution (Zhu et al., 2021). Activated mTORC1 phosphorylates the Ser/Thr residue (S211) of TFEB; this allows binding to the YWHA/14-three to three protein that restricts TFEB from nuclear translocation (Irazoqui, 2020). In contrast, the activation of calcineurin by mechanisms that increase the cytosolic concentration of Ca^2+^ induces dephosphorylation of the S211 residue in TFEB, thereby releasing TFEB and enabling its nuclear translocation. Subsequently, TFEB upregulates the CLEAR gene network and enhances autophagy and the biogenesis of lysosomes (Irazoqui, 2020). TFEB can also be directly dephosphorylated by protein kinase B (Akt) (S467), glycogen synthase kinase 3β (GSK3β) (S138), and extracellular regulated protein kinase 2 (ERK2) (S142), thus suggesting a complicated regulatory pattern of phosphorylation/dephosphorylation that balances the activation of TFEB (Zhu et al., 2021). In addition to phosphorylation, epigenetic mechanisms may also modulate the activity of TFEB (Zhu et al., 2021). For example, the deacetylation of TFEB enhances its activation in microglia cells (Bao et al., 2016) while ubiquitination degrades phosphorylated TFEB and subsequently enhances activity of the ALP (Sha et al., 2017). In contrast, the modification of TFEB by N6-methyladenosine (m^6^A) mRNA has been shown to reduce the expression of TFEB and impair the TFEB-dependent autophagic process (Song HL. et al., 2019). Other research has shown that TFEB is regulated by multiple positive feedback loops. TFEB possesses the CLEAR sequences within its promoter, thus implicating an autoregulatory mechanism (Martini-Stoica et al., 2016). Moreover, TFEB has been shown to upregulate the expression of mucolipin 1 (Mcoln-1), a calcium channel in the lysosomal membrane, to increase calcium efflux from the lysosomes and activate calcineurin; this also suggests the existence of a positive feedback loop (Martini-Stoica et al., 2016).

## TFEB and TFEB Dependent ALP in Sepsis

TFEB is an important pharmacological target in neurodegenerative, metabolic and malignant diseases, which was comprehensively summarized by previous literatures (Martini-Stoica et al., 2016; Raben and Puertollano, 2016). Meanwhile, recent studies have increasingly focused on its putative roles in immune modulation and organ protection, thereby implicating a close relationship with the development and outcomes of sepsis (Irazoqui, 2020; Zhu et al., 2021) (Figure 1).

### Modulation of the Immune Response

The cellular machinery of autophagy is featured by its ability to eliminate intracellular microbes in a process known as xenophagy (Deretic, 2021). In innate immune cells, TFEB is activated downstream of a phagocytosis event or can be stimulated by lipopolysaccharide (LPS) or interferon (IFN)-γ, thereby enhancing autophagy and lysosomal degradation and promoting the antimicrobial responses against intracellular pathogens (Kumar et al., 2020). It has also been demonstrated the antimicrobial activity is recovered through autophagy activation by overexpression of TFEB in sirtuin-3 KO macrophages (Kim et al., 2019). Of note, the membrane penetrating *Mycobacterium tuberculosis* was shown to repress the expression of *Tfeb* and inhibit autophagy in macrophages to support intracellular replication, implicating an underlying mechanism that disrupts xenophagy in sepsis (Ouimet et al., 2016). This may offer an alternative strategy for eliminating pathogens during sepsis by modulating the activity of TFEB.

The control of inflammation is another prominent effect of TFEB in sepsis. Several recent reviews have highlighted the functions of TFEB in modulating innate immunity and inflammation with wide diversity (Brady et al., 2018; Irazoqui, 2020). TFEB was shown to be rapidly activated in a *Caenorhabditis elegans* model following *Staphylococcus aureus* infection; this process drove the expression of proinflammatory cytokines and chemokines (Visvikis et al., 2014). The activation of TFEB has also been shown to induce M1 macrophage polarization, thus causing transition into a proinflammatory state (Pastore et al., 2016). In contrast, TFEB may exert anti-inflammatory effects indirectly by activating autophagy and lysosomes to suppress the activation of inflammasomes and the excessive production of proinflammatory cytokines (Irazoqui, 2020). TFEB also regulates lipid metabolism and restores homeostasis in the endoplasmic reticulum, thereby controlling the resolution of inflammation (Irazoqui, 2020). Therefore, TFEB may inhibit inflammation in sepsis *via* several different mechanisms.

Immunosuppression is also a well-recognized feature of sepsis and is characterized by monocytes/macrophage tolerance, T cell exhaustion, impaired antigen presentation and increased susceptibility to opportunistic nosocomial infection (Cecconi et al., 2018). Induction of the ALP may reverse these phenotypes (Venet and Monneret, 2018). Although there has been no direct report relating to TFEB and immunosuppression during sepsis, existing studies have demonstrated that TFEB intensifies immune responses by promoting antigen presentation in dendritic cells (Samie and Cresswell, 2015), resetting suppressive tumor-associated macrophages towards the M1 phenotype (Chen et al., 2018), reversing B cell senescence (Zhang et al., 2019), and by enhancing T cell immunity (Jin et al., 2021). Furthermore, the activation of TFEB mediates high levels of glucose-induced interleukin-1β secretion in human monocytic cells (Tseng et al., 2017) and rescues the dysfunctional lysosomal phenotype to improve viability in macrophages upon co-treatment with palmitate and LPS (Schilling et al., 2013). These findings also suggest that TFEB may reverse immunosuppression by restoring cellular response and preventing cell death.

### Organ Protection

The TFEB dependent ALP is fundamental for organ protection in sepsis by reducing tissue inflammation, alleviating oxidative stress and controlling metabolic process (Irazoqui, 2020). The overexpression of TFEB increases the autophagy levels in major organs and protects against LPS induce acute lung injury (Liu et al., 2019) or inflammatory liver injury and pancreatitis (Chao et al., 2018; Wang et al., 2019). Moreover, the pharmacological activation of TFEB by extrinsic stimuli or intrinsic signaling molecules is known to protect against septic liver injury, acute kidney injury (Li D. et al., 2021), and cardiac dysfunction (Unuma et al., 2013).

## The Pharmacological Application of TFEB Activators as a Therapeutic Option for Sepsis

Although the overexpression of TFEB has been investigated in cells and experimental rodents to evaluate its ability to activate the ALP, the pharmacological modulation of TFEB by means of activators is more desirable for therapeutic applications in the short term. For example, recent research has reported that small molecules and biomacromolecules (e.g., microRNAs (miRNA), polysaccharides and peptides) can modulate the ALP by activating TFEB (Xu H. et al., 2020; Chen et al., 2021). These activators have been categorized according to the pharmacological mechanisms by which they regulate TFEB. Here, we summarize recent findings related to the therapeutic implications of some of these molecules and biomacromolecules for sepsis.

### TFEB Activators and Their Pharmacological Targets

A variety of small compounds have been demonstrated to either increase the levels of TFEB or promote its activation at the post-translational level. The first target is the expression or synthesis of TFEB, thus leading to increased intracellular levels of TFEB. For example, peroxisome proliferator-activated receptor α (PPAR-α) agonists (e.g., GW7647 and cinnamic acid) and other natural compounds (e.g., genistein, ATRA, GDC-0941, and luteolin) have been shown to upregulate the mRNA expression of TFEB, thereby activating the ALP and enhancing the cellular clearance machinery (Enzenmuller et al., 2013; Moskot et al., 2014; Kim et al., 2017; Chandra et al., 2019; Xu H. et al., 2020; Orfali et al., 2020). Moreover, polyamines (e.g., spermidine) have been shown to activate translation factor eIF5A by inducing its hypusination, thereby promoting the synthesis of TFEB and the upregulation of autophagy in immune cells (Puleston et al., 2019; Zhang et al., 2019). The second target is the regulation of phosphorylation/dephosphorylation and translocation of TFEB in either a direct or indirect manner. For example, curcumin and analogues (e.g., curcumin-C1) are direct modulators that bind to the N terminus of TFEB, weaken TFEB-YWHA interaction, and promote the nuclear translocation of TFEB (Song et al., 2016; Zhang et al., 2016). The translocation of TFEB can be enhanced by the induction of dephosphorylation or the translation of TFEB in a manner that is mediated indirectly by mTOR inhibitors, AMPK/SIRT1 activators, ROS inducers, Akt inhibitors and Ca^2+^-calcineurin modulators (described in detail in Table 1).

**TABLE 1:** Small-molecular TFEB activators and their pharmacological targets.

Pharmacological targets	Name of compounds	References
TFEB expression	PPARα agonists (e.g., GW7647, cinnamic acid)	Kim et al. (2017), Chandra et al. (2019)
	Other compounds (genistein, ATRA, GDC-0941, luteolin)	Moskot et al. (2014), Orfali et al. (2020), Enzenmuller et al. (2013), Xu et al. (2020a)
TFEB synthesis	Polyamines (e.g., spermidine)	Zhang et al. (2019), Puleston et al. (2019)
TFEB binding	Curcumin and analogues (e.g., curcumin-c1)	Zhang et al. (2016), Song et al. (2016)
mTOR inhibition	Tool mTOR inhibitors (e.g. torin-1, rapamycin)	Xu et al. (2020b), Chen et al. (2021)
	Flavonoids (e.g., quercetin)	Huang et al. (2018)
	Polyphenols (e.g., 3,4-dimethoxychalcone, chlorogenic acid)	Chen et al. (2019b), Gao et al. (2020)
Ca^2+^/calcineurin modulation	Na^+^/K^+^-ATPase inhibitors (e.g., digoxin, Ouabain)	Wang et al. (2017), Song et al. (2019b)
	Other compounds (bedaquiline, liraglutide, carbon monoxide	Kim et al. (2018), Giraud-Gatineau et al. (2020), Fang et al. (2020b)
AMPK/SIRT1 activation	Resveratrol, licochalcone A	Huang et al. (2019), Lv et al. (2019)
Akt activation	Trehalose	Sharma et al. (2021)
ROS generation	Docetaxel, sulforaphane	Zhang et al. (2018), Li et al. (2021b)
TFEB dephosphorylation	Acacetin	Ammanathan et al. (2020)
TFEB nuclear translocation	Naringenin, Apigenin	Jin et al. (2017), Li et al. (2017)

In addition to small molecular activators, other larger biomolecules, such as polysaccharides, peptides and miRNAs, can also regulate the activity of TFEB. A few recent studies have reported that polysaccharides (e.g., tea polysaccharide and cyclodextrin) and peptides (e.g., apelin and cell-penetrating peptides) can activate TFEB and enhance the ALP process by inhibiting mTOR activity (Zhou et al., 2021) or promoting TFEB translocation (Song et al., 2014; Jiang et al., 2020; Kondow-Mcconaghy et al., 2020). In contrast, miRNAs are predominantly negative regulators of TFEB expression or its activity to promote autophagy and lysosome biogenesis. For example, a recent review article reported that specific miRNAs, such as the miR-128 and miR-29 families, are predicted to bind to the 3’ UTRs of TFEB mRNA, thereby downregulating its expression at the post-transcriptional levels (Cora et al., 2021). Other miRNAs such as miR-30b-5p (Guo et al., 2021) and miR-33 (Ouimet et al., 2016) have also been demonstrated to disrupt TFEB activation and inhibit autophagy by either inhibiting its transcriptional activity or interfering with its nuclear translocation. Therefore, downregulating these inhibitory non-coding RNAs may provide alternative approaches to activating the TFEB dependent ALP.

### Therapeutic Implications of TFEB Activators in Sepsis

Given that the impairment of ALP in sepsis results in unresolved infection, inflammation, organ injury, and immunodysfunction, researchers have begun to investigate TFEB modulators in preclinical models to examine their therapeutic efficacy in ameliorating sepsis-induced dysfunction by promoting autophagy and lysosomal functions.

TFEB-dependent xenophagy is critical for antimicrobial defense and is fundamental for the prevention and control of sepsis. TFEB-activating agents, such as trehalose (Sharma et al., 2021), bedaquiline (Giraud-Gatineau et al., 2020), and acacetin (Ammanathan et al., 2020), can upregulate autophagy- and lysosome-associated genes, thus resulting in enhanced bactericidal activity in macrophages or mice infected by intracellular pathogens (e.g., *Mycobacterium tuberculosis* and *Salmonella typhimurium*). It is notable that opportunistic pathogens, such as mycobacterial species, can commonly induce secondary infection in post-sepsis patients or trigger sepsis in patients who are immunocompromised. Moreover, pathogens like *Mycobacterium tuberculosis* inhibit autophagy and lysosomal function by inducing miR-33 dependent inactivation of TFEB (Ouimet et al., 2016). Therefore, TFEB activators may provide additional benefit in these circumstances.

The TFEB-dependent ALP is widely regarded as a critical pathway that can control excessive inflammation and restore immune homeostasis during sepsis. Several recent studies have described pivotal immunomodulatory roles for TFEB activators in sepsis. The activation of TFEB by flavonoid compounds, such as naringenin and apigenin, has been shown to reduce inflammatory cytokines following an LPS challenge by either enhancing the degradation of intracellular cytokines *via* lysosome-dependent mechanisms (Jin et al., 2017) or by enhancing the control of autophagy (Li et al., 2017). Two recent studies reported that polyamines can promote the synthesis of TFEB and induce autophagy; this ability was successfully used to reverse immune senescence in B lymphocytes (Zhang et al., 2019) or to promote macrophage M2 polarization (Puleston et al., 2019). These findings further indicate that TFEB activators may also modulate the status of immune cells in addition to their anti-inflammatory activity *via* a common mechanism to activate the ALP.

TFEB activators have also been demonstrated to confer organ protection in sepsis. For example, licochalcone A, curcumin, and cobalt protoporphyrin, have been shown to alleviate liver injury (Lv et al., 2019), intestinal barrier injury (Cao et al., 2020), and septic insults in the heart (Unuma et al., 2013), induced by LPS challenge; these effects were all related to the ability of these TFEB activators to activate the ALP. Other non-conventional TFEB inducers, such as hydrogen rich saline (Fu et al., 2020) and carbon monoxide (Kim et al., 2018), have been found to confer protection against lipopolysaccharide-induced acute injury or inflammatory liver injury, respectively. Furthermore, the activation of TFEB by mTORC1 inhibitors has been shown to rescue a mouse model from lethal pancreatitis (Wang et al., 2019) and chronic ethanol-induced liver injury (Chao et al., 2018); these effects were closely associated with the onset of sepsis or during sepsis and worsened clinical outcomes.

## Conclusion and Perspectives

A multitude of studies have aimed to explore the therapeutic efficacy of TFEB and the TFEB-dependent ALP pathway in sepsis *via* the pharmacological modulation of TFEB activation. This research has led to encouraging outcomes in preclinical models of sepsis or diseases associated with sepsis, including antimicrobial, anti-inflammatory, and organ protective activities. In addition, lower nuclear levels of TFEB have been detected in patients with alcohol-induced hepatitis or acute pancreatitis. These results further implicate the significance of activating TFEB and the TFEB-dependent ALP in clinical conditions associated with sepsis (Chao et al., 2018; Wang et al., 2019). However, despite these encouraging findings, there are still several concerns that need to be addressed in future research activities.

First, the kinetics and specificity of TFEB in sepsis should be investigated. Both the activity of TFEB and the status of autophagy are altered during the progression of sepsis. Therefore, further investigations are needed to evaluate time-dependent changes in the expression and activity of TFEB in sepsis; these studies are vital if we are to use TFEB activators appropriately for therapeutic intervention. In addition, it is known that the dephosphorylation/phosphorylation balance of TFEB is central for its regulation. Multiple pathways, such as mTORC1 and Ca^2+^/calcineurin, target the phosphorylating sites on TFEB. However, these mechanisms are identified in different cells. Further research is needed to find the specific upstream regulatory pathways in each cell type and determining their function of modulating TFEB in sepsis (Pan et al., 2017; Pan et al., 2020). Moreover, most existing studies investigated the activation of TFEB by assessing the efficacy of TFEB activators in cell lines or homogenates from a single organ. The entire tissue- and organ-specific profiles of TFEB and the TFEB-dependent ALP in sepsis have yet to be elucidated in detail. In this regard, it is important that we investigate the overall activity of TFEB and the TFEB-dependent ALP in major organs (e.g., liver, lung, spleen, kidney and intestine) or specific cells (e.g., immune cells, hepatocytes and endothelial cells). It is also important to compare the involvement of TFEB and the TFEB-dependent ALP in organ-specific functions (e.g., hepatic metabolic regulation, cardiac and renal protection, and immunomodulation) to determine their influences on the outcomes of sepsis.

Second, the activation of the TFEB-dependent ALP can be triggered by nutritional deficiency, infection, and other stressful conditions, that are commonly observed in sepsis. Thus, future studies need to investigate whether TFEB activators can synergize or counteract with these environmental factors in the regulation of TFEB and the ALP. Prolonged deficiency or dysregulation in sepsis is increasingly regarded as an epigenetic consequence (van der Poll et al., 2017) while post-translational mechanisms may also be key to the activation of TFEB and how the ALP is affected over the long term. Therefore, further studies should focus on the epigenetic regulation of TFEB and identify post-translational mechanisms (e.g., miRNAs, long non-coding RNA (lncRNAs) and new protein modifications) that may exert functional roles during sepsis.

Third, the current safety and efficacy data for TFEB activators are not convincing and require further validation. For example, the efficacy of TFEB activators should be verified in more standardized models of sepsis (e.g., the cecal ligation and puncture model) instead of endotoxemia models or otherwise tested in clinical settings. Moreover, most TFEB activators target the upstream regulators of TFEB, such as mTORC1 and Ca^2+^/calcineurin; these are also involved in other intracellular events (Song et al., 2021). Therefore, more direct TFEB activators (e.g., curcumin-c1) need to be developed in future studies as these may increase functional specificity and reduce side effects. In addition, we must consider that the activation of the TFEB-dependent ALP may induce tumorigenesis, a disease condition that is frequently concurrent with sepsis (Levine and Kroemer, 2019). Therefore, it is important that we evaluate the relative risks and benefits before the widespread therapeutic application of TFEB activators by investigating their consequences in septic conditions concomitant with tumor.
